# Transforming growth factor-β1 promotes breast cancer metastasis by downregulating miR-196a-3p expression

**DOI:** 10.18632/oncotarget.16308

**Published:** 2017-03-17

**Authors:** Yan Chen, Shai Huang, Bo Wu, Jiankai Fang, Minsheng Zhu, Li Sun, Lifeng Zhang, Yongsheng Zhang, Maomin Sun, Lingling Guo, Shouli Wang

**Affiliations:** ^1^ Department of Surgery, The First Affiliated Hospital of Soochow University, Suzhou 215006, China; ^2^ Department of Pathology, School of Biology & Basic Medical Sciences, Soochow University, Suzhou 215123, China; ^3^ Department of Surgery, The People's Hospital of Sihong County, Sihong 223900, Jiangsu Province, China; ^4^ Laboratory Animal Research Center, Soochow University School of Medicine, Suzhou 215123, China; ^5^ Department of Pathology, The Second Affiliated Hospital of Soochow University, Suzhou 215004, China; ^6^ Laboratory of Molecular Pathology, Soochow University & Sihong County People's Hospital, Suzhou 215123, China; ^7^ Suzhou Key Laboratory of Tumor Microenvironment and Pathology, Soochow University, Suzhou 215006, China

**Keywords:** microRNA, miR-196a-3p, transforming growth factor-β1, breast cancer

## Abstract

Transforming growth factor-β1 is considered a key contributor to the progression of breast cancer. MicroRNAs are important factors in the development and progression of many malignancies. In the present study, upon studies of breast cancer cell lines and tissues, we showed that microRNA -196a-3p is decreased by transforming growth factor-β1 in breast cancer cells and associated with breast cancer progression. We identified neuropilin-2 as a target gene of microRNA -196a-3p and showed that it is regulated by transforming growth factor-β1. Moreover, transforming growth factor-β1-mediated inhibition of microRNA -196a-3p and activation of neuropilin-2were required for transforming growth factor-β1-induced migration and invasion of breast cancer cells. In addition, neuropilin-2 expression was suppressed in breast tumors, particularly in triple-negative breast cancers. Collectively, our findings strongly indicate that microRNA -196a-3p is a predictive biomarker of breast cancer metastasis and patient survival and a potential therapeutic target in metastatic breast cancer.

## INTRODUCTION

Despite considerable progress in the early diagnosis and surgical therapy of breast cancers, breast cancer is the second leading cause of death in the United States [1], and the first cause of cancer-related death in women younger than 45 years followed by lung cancer in China [2]. Transforming growth factor-β1 (TGF-β1) is considered key contributor to the progression of breast cancer [3, 4]; TGF-β1 signaling features a growth inhibitory effect at an early stage of breast cancer cells, but aggressive oncogenic activity at the advanced malignant state [5–7]. Recent results indicate an association between TGF-β1 signaling and microRNAs (miRNAs, miR), as revealed by our previous studies in rhabdomyosarcoma [8, 9] and those of others about breast cancers [4, 10–13], providing new insight into the nature of cancer.

MiRNAs, which are noncoding RNAs that regulate target gene expression post-transcriptionally, show unbalanced expression in cancers [14], and there is increasing evidence of the efficacy of miRNA-based therapies [15]. Considering that TGF-β1 promotes the progression of breast cancer at advanced stages [3, 4], we assumed that miRNA expression inhibited by TGF-β1 must have antitumor potential, as previously demonstrated for miR-450b-5p [8] and miR-411-5p [9]. Using TGF-β1 knockdown colorectal cancer cells (HT-29, SW620 and LoVo) and comprehensive locked nucleic acid microarray analyses, five significantly raised miRNAs were selected (Tracking Number: GSE53338, unpublished data), and their expression was examined in breast cancer. We found that TGF-β1 suppressed miR-196a-3p in colorectal cancer and in breast cancer and exerted an antitumor effect on breast cancer.

In the present study, we showed that ectopic overexpression of miR-196a-3p reversed TGF-β-induced breast cancer cell migration and invasion. We also identified the protein neuropilin 2 (NRP2) as a downstream target of miR-196a-3p, and showed that blocking NRP2 expression potently antagonized TGF-β promigratory effects in invasive breast cancer cells. Collectively, our results demonstrate that miR-196a-3p and its downstream target NRP2 are involved in TGF-β-induced cell migration and invasion in invasive breast cancer cells, suggesting that restoring the expression of miR-196a-3p is a potential therapeutic approach for the treatment of breast cancer.

## RESULTS

### miR-196a-3p expression is reduced in breast cancer in correlation with metastatic potential

**Figure 1:**
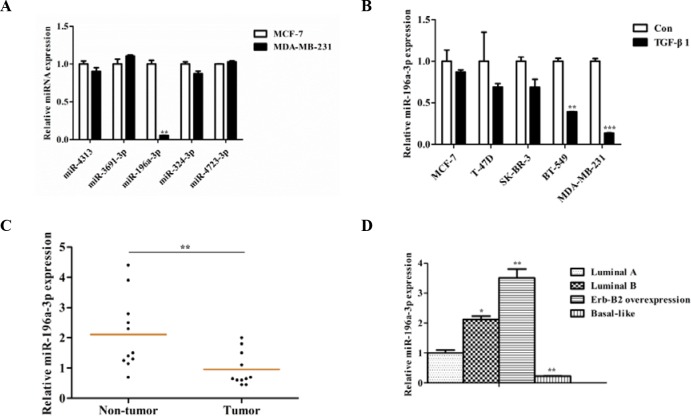
miR-196a-3p is reduced in breast cancer cell lines and clinical specimens **(A)** The expression of TGF-β1-inhibited miRNAs was evaluated by qRT-PCR in breast cancer cell lines with different metastatic potentials. The results are means of three independent experiments ± S.D. (**p< 0.01). **(B)** qRT-PCR analysis of the effect of TGF-β1 on miR-196a-3p expression in breast cancer cell lines with different metastatic potentials stimulated with or without 5 ng/ml TGF-β 1for 24 h. The results are means of three independent experiments ± S.D. (**p< 0.01,***p< 0.001). **(C)** qRT-PCR analysis of the mRNA expression of miR-196a-3p in 13 human breast cancer tissues and 11 normal breast samples. **(D)** miR-196a-3p expression in 12 different kinds of breast cancers (3 luminal A, 3 luminal B, 3 ERBB2 overexpressing, and 3 basal-like cases). The results are means of three independent experiments ± S.D. (*p<0.05,**p< 0.01).

In a previous study, we identified five TGF-β1 inhibited miRNAs in colorectal cancer by microarray analysis (GSE53338), including miR-4313, miR-3691-3p, miR-196a-3p, miR-324-3p, and miR-4723-3p. To investigate whether these miRNAs are correlated with the malignant behavior of breast cancer, we examined the expression of these miRNAs in two different breast cancer cell lines (the noninvasive luminal cell line MCF-7 and the highly invasive cell line MDA-MB-231) by reverse transcription-polymerase chain reaction(RT-PCR). We found that only miR-196a-3p was markedly decreased in MDA-MB-231 cells (Figure 1A). To further verify the relationship between miR-196a-3p expression and the malignant behavior of breast cancer, three noninvasive breast cancer cell lines (MCF-7, T-47D, and SK-BR-3) and two highly invasive breast cancer cell lines (BT-549 and MDA-MB-231) were analyzed. We found that miR-196a-3p was inhibited by TGF-β1 in all tested breast cancer cell lines, and the expression of miR-196a-3p was higher in noninvasive breast cancer cell lines than in the two highly invasive breast cancer cell lines (Figure 1B).

We then detected miR-196a-3p expression levels in 13 primary tumors and 11 non-cancerous specimens. As shown in Figure 1C, miR-196a-3p was declined by approximately two-fold in tumors compared with non-cancerous specimens. Moreover, miR-196a-3p expression was lower in basal like breast cancer than in the other subtypes of breast cancer (Figure 1D). Taken together, these results suggest that miR-196a-3p plays an important role downstream of TGF-β-signal transduction in breast cancer.

### Decreased expression of miR-196a-3p correlates with poor clinical outcomes in breast cancer

The expression of miR-196a-3p was examined using real-time qRT-PCR in 112 breast cancer tissue samples and the association of miR-196a-3p expression levels with clinicopathological features was analyzed. As shown in Table 1, low miR-196a-3p expression levels were closely correlated with lymph node metastasis (*p*=0.008), TNM stage (*p*=0.001), and pathological differentiation (*p*=0.029). However, no significant correlations between miR-196a-3p expression and age, tumor size, estrogen receptor (ER) status, progesterone receptor (PR) status and human epidermal growth factor receptor 2 (HER2) status were found. Our results indicate that miR-196a-3p may play a critical part in the carcinogenesis and progression of breast cancer.

**Table 1:** Relationship between miR-196a-3p expression and clinicalpathologic variables of breast cancer patients

Parameters	Number of cases	miR-196a-3p expression		*P* value
		Low (52)	High (60)	
**Age(years)**				
<50	49	18	31	0.07
≥50	63	34	29	
**Tumor size(cm)**				
<2	63	25	38	0.105
≥2	49	27	22	
**TNM stage**				
I+II	70	24	46	0.001*
III	42	28	14	
**Differentiation grade**				
G1+G2	84	34	50	0.029*
G3	28	18	10	
**Lymph node metastasis**				
Yes	56	33	23	0.008*
No	56	19	37	
**ER status**				
Negative	58	32	26	0.054
Positive	54	20	34	
**PR status**				
Negative	57	30	27	0.18
Positive	55	22	33	
**HER2 status**				
Negative	57	31	26	0.086
Positive	55	21	34	

* *P* < 0.05 was considered significant.

**Figure 2:**
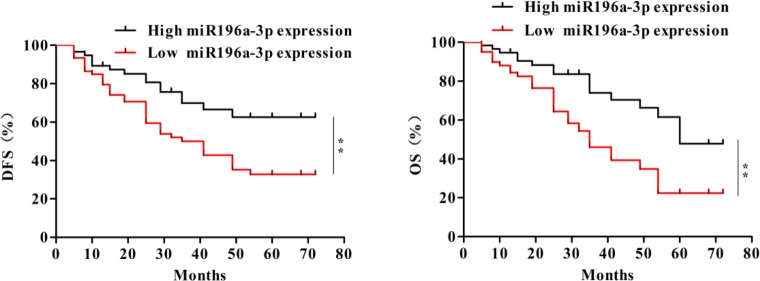
Decreased expression of miR-196a-3p correlates with poor clinical outcomes in breast cancer The overall survival (OS) (left) and disease-free survival (DFS) (right) curves for the studied patients with high or low miR-196a-3p expression,**p< 0.01.

To further investigate the significance of miR-196a-3p in terms of clinical prognosis, the Kaplan–Meier method and log-rank test were used to evaluate the differences in overall survival and disease-free survival between the low-expression group and the high-expression group. As shown in Figure 2, the 5-year overall survival of the low-miR-196a-3p expression group was significantly shorter than that of the high-miR-196a-3p expression group (log rank test, *p*=0.0019). Moreover, patients with low expression of miR-196a-3p had significantly shorter disease-free survival than patients with high miR-196a-3p expression (log rank test, *p*=0.0063). These results indicated that inhibition of miR-196a-3p expression may be correlated with poor survival in breast cancer patients.

### miR-196a-3p suppresses TGF-β-mediated migration and invasion

**Figure 3:**
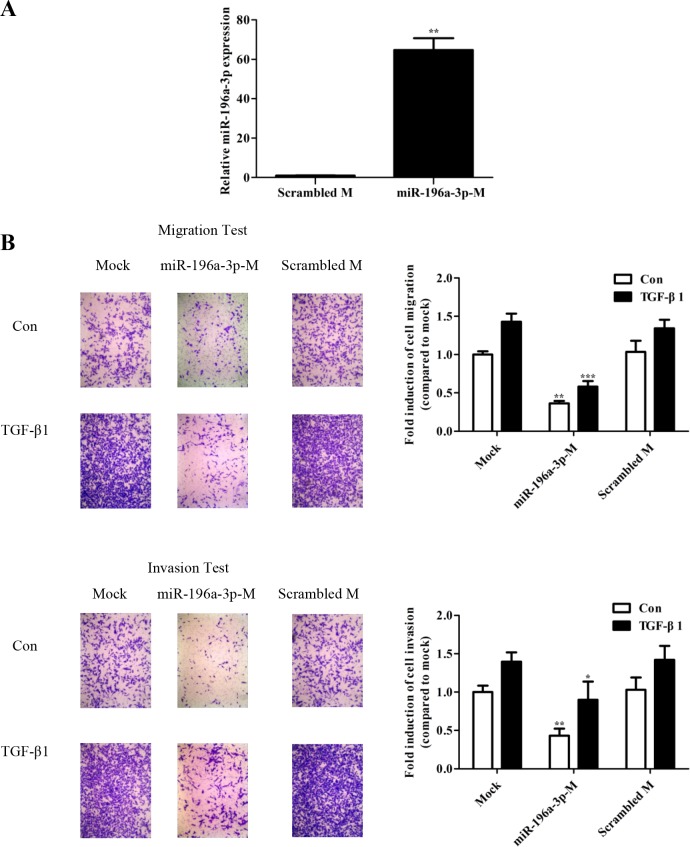
miR-196a-3p suppresses TGF-β1-mediated migration and invasion in breast cancer **(A)** qRT-PCR analysis of miR-196a-3p levels in MDA-MB-231 cells transfected with 80 nM mimic. The results are means of three independent experiments ± S.D. (**p< 0.01). **(B)** MDA-MB-231 cells were transfected with 80 nm miR-196a-3p mimic. The migratory and invasive behavior was measured at 24 h after stimulation with 5 ng/ml TGF-β1.(miR-196a-3p-M: miR-196a-3p-mimics; Scrambled M: Scrambled mimics; Con: Control) The results are means of three independent experiments ± S.D. (*p<0.05,**p< 0.01,***p< 0.001).

TGF-β1 was shown to promote cellular migration and invasion in human breast cancer cells [16–18]. To investigate whether TGF-β1-inhibited miR-196a-3p bearing effect of anti-tumor on breast cancer, we tested miR-196a-3p-mimics. Transient transfection of 80 nM mimic into MDA-MB-231 cells resulted in an approximately 60-fold increase of miR-196a-3p (Figure 3A). Cell migration and invasion were assessed by the Transwell assay in MDA-MB-231 cells transfected with the miR-196a-3p mimic or a scrambled mimic as a negative control and stimulated with or without TGF-β. The number of cells migrated through the Transwell was calculated after 24 h. As shown in Figure 3B, TGF-β induced MDA-MB-231 cell migration and invasion in the mock and control groups, whereas the miR-196a-3p mimic-treated groups showed no significant difference in the metastatic ability of MDA-MB-231 cells with or without TGF-β1 stimulation. In addition, the overexpression miR-196a-3p did not affect the proliferative properties of breast cancer cells as determined by the MTT assay (Supplementary Figure 1).

### NRP2 is the target gene of miR-196a-3p and is regulated by TGF-β1

**Figure 4:**
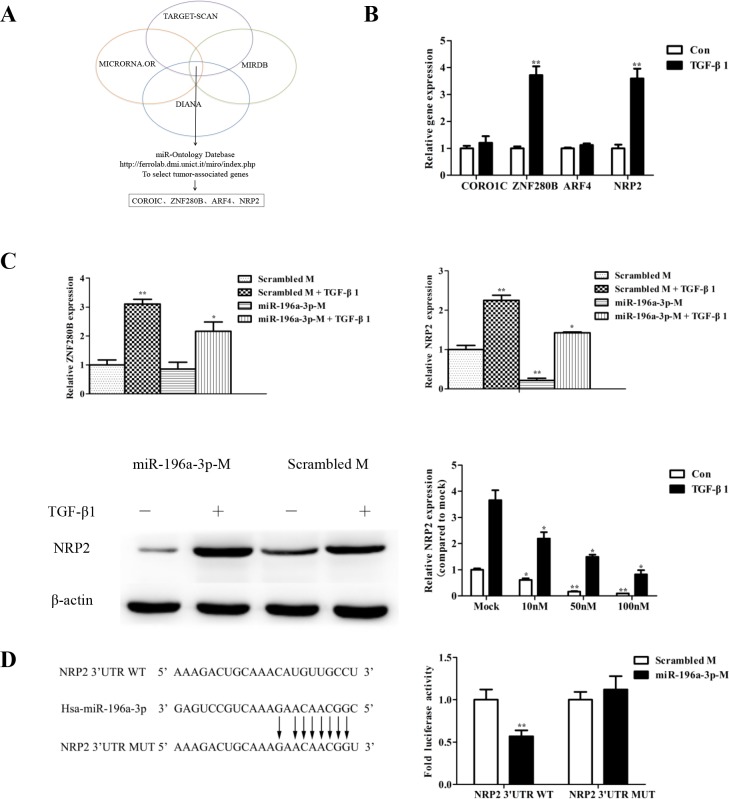
NRP2 is the target gene of miR-196a-3p and is regulated by TGF-β1 **(A)** Bioinformatics algorithms identified the top four potential predicted target genes of miR-196a-3p. **(B)** Total RNA was extracted from MDA-MB-231 cells that were stimulated with 5 ng/ml TGF-β1 for 24 h, and the miRNA expression of target candidates was measured by qRT-PCR. The results are means of three independent experiments ± S.D. (**p< 0.01,). **(C)** MDA-MB-231 cells were transfected with 80 nM negative control mimic and miR-196a-3p mimic and stimulated with or without 5 ng/ml TGF-β1 for 24 h. The expression of target gene candidates was measured by qRT-PCR(miR-196a-3p-M: miR-196a-3p-mimics). The results are means of three independent experiments ± S.D. (*p<0.05, **p< 0.01). **(D)** MDA-MB-231 cells were transiently transfected with a Renilla luciferase reporter gene whose expression was driven by either wild-type or mutant (mut) NRP2–3′-UTR seed sequences, and a luciferase assay was performed. The results are means of three independent experiments ± S.D. (**p< 0.01).(B∼C: Con: Control).

Bioinformatics analyses using various algorithms such as DIANA, MICRORNA.OR, and MIRDB identified the top four potential predicted targets for miR-196a-3p (Figure 4A). Only two target genes, ZNF280B and NRP2, were increased in response to TGF-β stimulation (Figure 4B). To investigate whether the TGF-β-mediated activation of ZNF280B and NRP2 was miR-196a-3p-dependent, we examined the effect TGF-β stimulation on the levels of ZNF280B and NRP2 in MDA-MB-231 cells transfected with or without the miR-196a-3p mimic. The results showed that the miR-196a-3p mimic had no effect on ZNF280B levels, whereas it significantly inhibited the expression of NRP2 and reversed the effect of TGF-β1 on the expression of NRP2 (Figure 4C).

Moreover, transient transfection of a NRP2-3′-UTR-luciferase reporter into MDA-MB-231 cells stably expressing miR-196a-3p showed that miR-196a-3p decreased luciferase activity in the NRP2-3′-UTR construct. Importantly, mutation of the miR-196a-3p seed sequence located within the NRP2-3′-UTR prevented miR-196a-3p from suppressing luciferase expression in MDA-MB-231 cells (Figure 4D). These results indicated that miR-196a-3p inhibits NRP2 expression by binding to its complementary seed sequence in the NRP2-3′-UTR, suggesting that NRP2 is a target gene of miR-196a-3p and is controlled by the TGF-β1 signaling pathway.

### NRP2 promotes TGF-β-mediated migration and invasion

**Figure 5:**
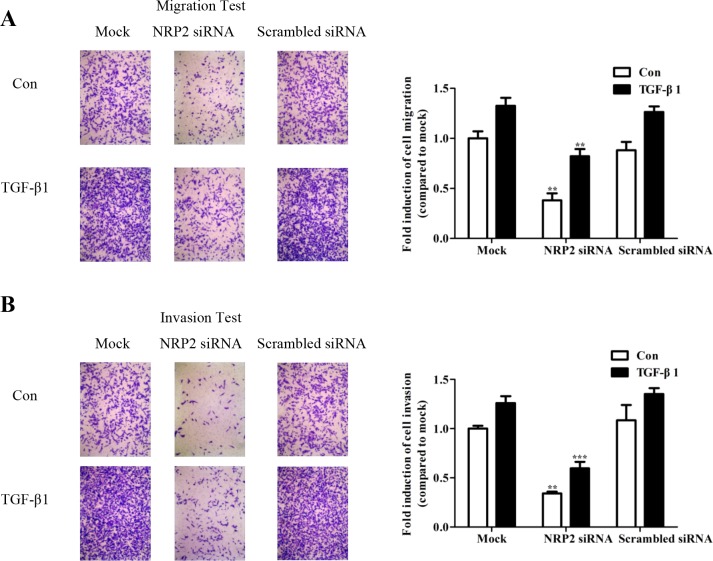
NRP2 promotes TGF-β1-mediated migration and invasion MDA-MB-231 cells were transfected with 80 nm NRP2 siRNA and siRNA negative controls, the migratory **(A)** and invasive **(B)** behaviors were measured at 24 h after stimulation with 5 ng/ml TGF-β1 The results are means of three independent experiments ± S.D. (**p< 0.01,***p< 0.001). (Con: Control).

Toinvestigate whether NRP2 expression is required for TGF-β-mediated cell migration and invasion, NRP2 was silenced using an siRNA specific to human NRP2 (Supplementary Figure 2). Knockdown of NRP2 expression significantly inhibited TGF-β1-induced migration (Figure 5A,) and invasion (Figure 5B) of MDA-MB-231 cells. The effects of the NRP2 siRNA on the viability of breast cancer cells was examined using the MTT assay, which showed that NRP2 silencing did not change the proliferative properties of the cells (Supplementary Figure 3), consistent with effect of the miR-196a-3p mimic. These results indicate that miR-196a-3p and NRP2 are involved the same pathway downstream of TGF-β to manipulate cellular migration and invasion in breast cancer cells.

### miR-196a-3p inhibits the proliferation and metastasis of MDA-MB-231 cells *in vivo*

**Figure 6:**
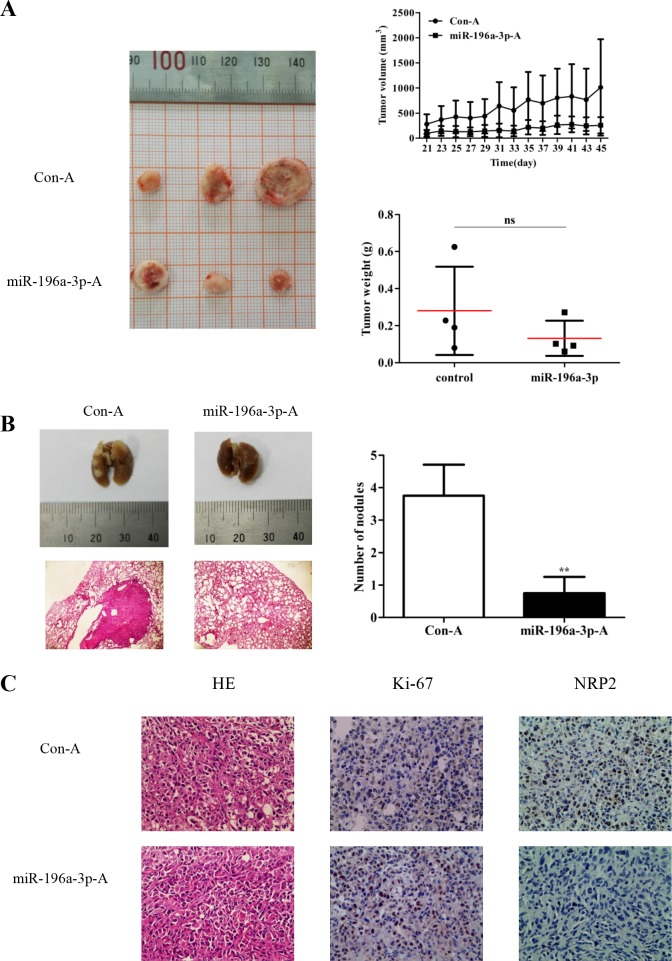
miR-196a-3p inhibits the proliferation and metastasis of MDA-MB-231 cells *in vivo* **(A)** Nude mice were challenged in the mammary fat with MDA-MB-231 cells to form xenograft tumors, and miR-196a-3p or control agomir was injected into the tumors. Tumor volumes and weights were monitored. ns:nonsignificance, *p*=0.1662. **(B)** MDA-MB-231 cells were randomly injected into nude mice via tail vein, followed by administration of miR-196a-3p or control agomir. Metastatic nodules in the lung were observed. Representative lungs were photographed and HE staining of lungs was performed. The results are means of three independent experiments ± S.D. (**p< 0.01). **(C)** Immunohistochemical staining of Ki67 and NRP2 in xenograft tumor tissues. **p<0.01 (×200). (miR-196a-3p-A: miR-196a-3p-agomir; Con-A:Control agomir)

The mammary fat pads of nude mice were inoculated *in situ* with MDA-MB-231 cells. When the xenograft tumors reached 50 mm^3^, miR-196a-3p and control agomir were injected into the tumors (1 nmol/injection, once every 2 days). Mice were sacrificed at 24 days after the first injection. As shown in Figure 6A, both the volume and weight of tumors were significantly lower in miR-196a-3p-mimic treated groups than in control groups. To investigate the effect of miR-196a-3p on the metastasis of breast cancer cells, MDA-MB-231 cells were randomly injected into nude mice via the tail vein, followed by administration of miR-196a-3p and control agomir. Metastatic nodules were observed on the surface of the lungs in mice injected with control agomir, whereas no nodules were found in mice injected with miR-196a-3p agomir (Figure 6B). Immunostaining analysis showed that miR-196a-3p inhibited ki-67 expression and NRP2 expression (Figure 6C). These data indicated that miR-196a-3p inhibited the metastasis of MDA-MB-231 cells by targeting NRP2 *in vivo*.

### NRP2 expression is negatively related to the expression of miR-196a-3p in breast cancers, especially in triple-negative breast cancer(TNBC)

**Figure 7:**
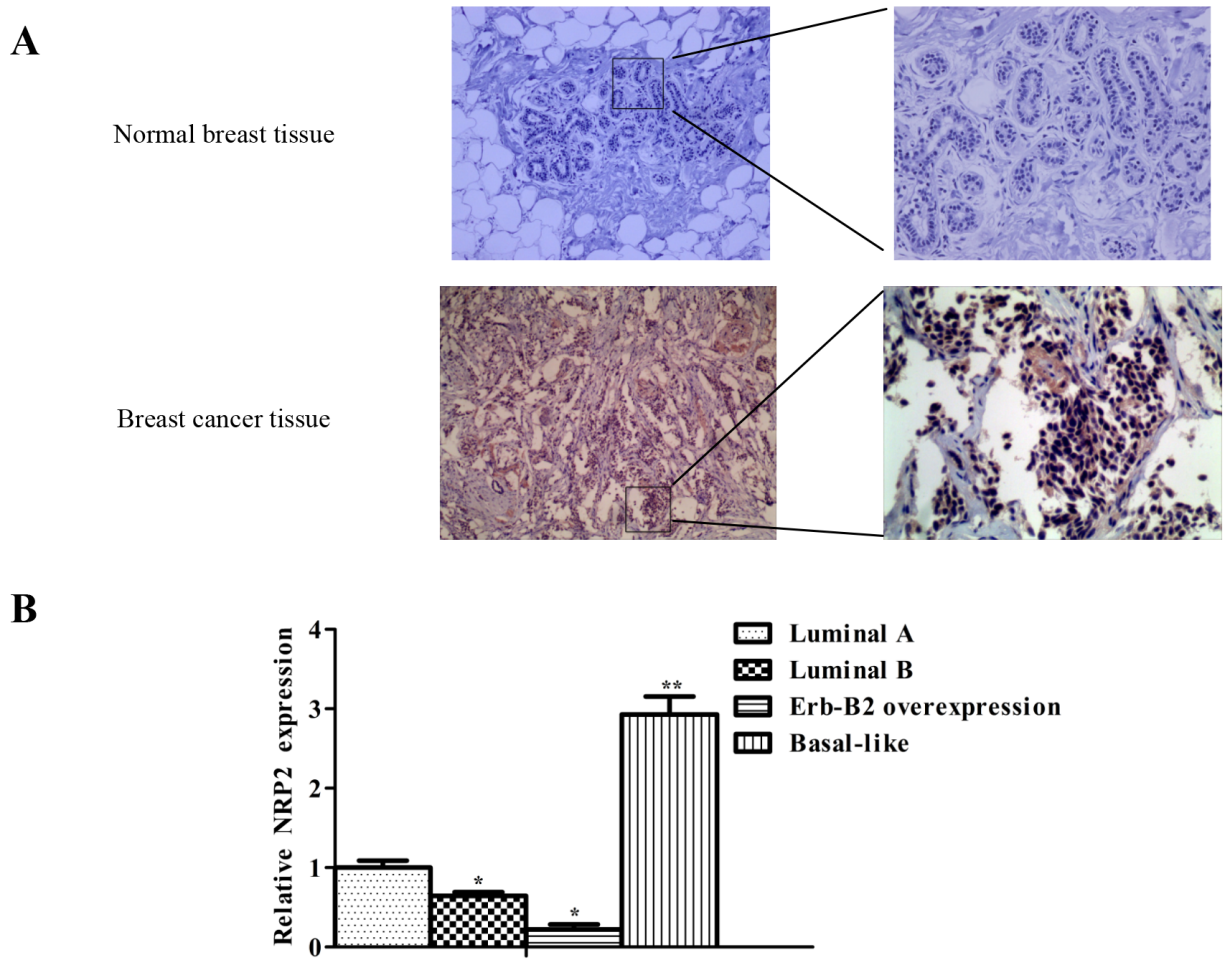
NRP2 expression is negatively related to the expression of miR-196a-3p in breast cancers **(A)** NRP2 immunostaining of primary tumors and corresponding non-tumor tissues(Left, ×100; Right, ×200). **(B)** NRP2 expression in 12 different kinds of breast cancers was measured by qRT-PCR. (3 luminal A, 3 luminal B, 3 ERBB2 overexpressing, and 3 basal-like cases). The results are means of three independent experiments ± S.D. (*p<0.05,**p< 0.01).

NRP2 protein levels were examined by immunostaining and comprising 10 cases of paired non-tumor and primary breast cancer tissues. The results showed that higher levels NRP2 in membrane and cytoplasimic was detected in all primary breast cancer tissues than in non-tumor (Figure 7A). In addition, the level of NRP2 expression was negatively related to the expression of miR-196a-3p in breast cancers, especially in TNBC (Figure 7B). These results provided additional evidence supporting the theory that NRP2 acts downstream of miR-196a-3p in breast cancers.

## DISCUSSION

**Figure 8:**
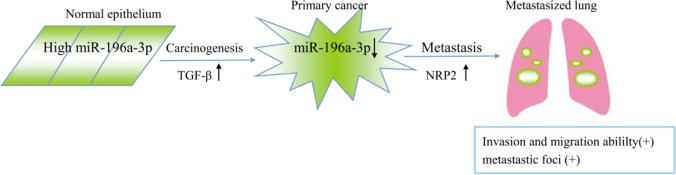
Proposed model of the role of miR-196a-3p in TGF-β-induced cell migration mediated by targeting NRP2 in breast cancer

Metastasis is the primary cause of breast cancer mortality [19, 20], and miRNAs are involved in regulating the behavior of tumor metastasis [21, 22]. Here, we proposed a model in which TGF-β is capable of inducing tumorigenic through the reduction of miR-196a-3p, which targets NRP2, leading to its accumulation (Figure 8), providing novel potential targets for the treatment of metastatic breast cancers.

It is well established that TGF-β has a great deal of significance in the progression of breast cancer [3, 23]. Recently, microRNAs were involved in multiple TGF-β-mediated processes that eventually lead to tumor metastasis [10]. Based on our previous finding that TGF-β1-related miRNAs contribute to tumor progression [8, 9, 24], we explored TGF-β1 inhibited miRNAs in colorectal cancer cells, including miR-4313, miR-3691-3p, miR-196a-3p, miR-324-3p, and miR-4723-3p, and demonstrated that only miR-196a-3p was restrained in breast cancer lines. Furthermore, miR-196a-3p expression was lower in breast cancer tissues than in adjacent normal breast tissues and associated with lymph node-metastasis, clinical stage, and pathological differentiation. We thus propose that blocking miR-196a-3p expression may be a prerequisite to TGF-β-mediated migration and invasion, as assessed by Transwell assays.

Reports on miR-196a-3p function are rare. Here, to determine the mechanism underlying the antimetastatic activity of miR-196a-3p, we selected four potential targets using available online algorithms, and only two target genes were affected under the stimulation of TGF-β1. Based on the mRNA and protein expression of the two candidate target genes in response to miR-196a-3p mimics, we focused on NRP2 as a target gene of miR-196a-3p in breast cancer. NRP2 expression was silenced using a specific siRNA, which inhibited TGF-β-mediated migration and invasion, as shown in Figure 5. This suggested that NRP2 expression modulated by miR-196a-3p has an effect in influencing specific steps in the metastatic cascade. Based on our finding that miR-196a-3p mimics inhibit the metastasis of breast cancer cells, which was reinforced by knockdown of NRP2 and antagonized by TGF-β1 stimulation, and previous reports that NRP2 acts as an oncogene to promote metastasis in cancers such as breast cancer [25], esophageal squamous cell carcinoma [26], and melanoma [27], we hypothesized that miR-196a-3p and its downstream target NRP2 is involved in TGF-β-induced cell migration and invasion in breast cancer. The present study provides evidence confirming the crosstalk between the TGF-β signaling pathway and miRNAs in breast cancer.

The multifunctional cytokine TGF-β1 has a direct influence on breast cancer progression. In the early stages of breast cancer, TGF-β1 inhibits epithelial cell cycle progression and shows tumor suppressive effects. However, in the late stages, TGF-β1 is linked with increased tumor progression and metastasis [3]. Although several studies showed crosstalk between TGF-β1 signaling and miRNAs in breast cancer [4, 10], the role of TGF-β1 signaling in breast cancer remains unclear. Many effects of TGF-β1 on the progression of breast cancer remain undetermined, such as epithelial-to-mesenchymal transition [28, 29], the crucial function of TGF-β1 as a modulator of matrix metalloproteinases [6, 30], and TGF-β1 mediated recruitment of tumor microenvironment [24, 31]. The relationship between the TGF-β signaling pathway and miRNAs should be explored further in clinical studies.

Recently, there are studies found that miR-196 can promote tumor invasion and proliferation through different signaling pathways in a variety of tumors [32, 33], and it is becoming an emerging cancer biomarker for digestive tract cancers [34]. Furthermore, the NRP2 can promotes tumourigenicity and metastasis in oesophageal squamous cell carcinoma through ERK–MAPK–ETV4–MMP–E-cadherin deregulation [26] and it is uniquely support TGF-β-mediated progression in lung cancer [35]. All of which laid the foundation for the specific molecular mechanism of TGFβ1-miRNA-NRP2 this axis for promoting breast cancer metastasis, and provide an important molecular target for breast cancer treatment.

## MATERIALS AND METHODS

### Cell lines and cell culture

The human breast cancer cell lines MCF-7, T47D, SK-BR3, BT-549 and MDA-MB-231 were obtained from American Type Culture Collection (ATCC; Manassas, VA, USA). MCF-7 and BT-549 cells were cultured in RPMI-1640 medium. T47D, SK-BR3, and MDA-MB-231 cells were maintained in Dulbecco's modified Eagle's medium(DMEM). Both culture media were supplemented with 10% fetal bovine serum (FBS), 2 mM L-glutamine, 100 units/ml penicillin, and 100 μg/ml streptomycin. The cells were cultured at 37°C in a humidified atmosphere containing 5% CO_2_.

### Patients and tissue samples

A total of 112 breast cancer tissues and corresponding noncancerous breast tissues were collected directly from surgery after removal of the necessary amount of tissue for routine pathology examination at the Department of Pathology, Second Affiliated Hospital of Soochow University, between 2008 and 2014. The tissues were immediately frozen in liquid nitrogen after surgical removal and stored at −80°C until use. None of these patients had received radiotherapy or chemotherapy prior to surgery. All the specimens were diagnosed by pathologists. Informed written consent was obtained from all patients. Patients' clinical information was stored in a database and summarized in Table 1.

### Bioinformatics search

Potential targets were predicted by performing a search in the following online databases: Diana microT4.0 (http://diana.imis.athena-innovation.gr/DianaTools/index.php?r=microT_CDS/index), MICRORNA.OR (http://www.microrna.org/microrna/home.do) and MIRDB (http://mirdb.org/miRDB/).

### Transfections

Cells were cultured to 90% confluence in six-well plates and transfected with 80 nM siRNA or mimic(Ribobio Co., Guangzhou, China). Transfections were performed using 5 μl Lipofectamine 2000 (Invitrogen, Carlsbad, CA, USA) in 500 μl Opti-MEM (Gibco-BRL, Grand Island, NY, USA) with the desired concentration of miRNA or siRNA. Cells were incubated for 6 h with transfection reagents in starvation medium. Then, cells were stimulated with 5 ng/ml [36] TGF-β1 for the indicated times.

### Reverse transcription-polymerase chain reaction

Cells were transfected with or without siRNA or mimics and treated with or without 5 ng/ml TGF-β1 (PeproTech Inc., Rocky Hill, NJ, USA) for different times. Total RNA was extracted with the TRIzol reagent (Tiangen Biotech, Beijing, China). Reverse transcription was carried out using M-MLV RT (Invitrogen, Carlsbad, CA, USA) according to the manufacturer's instructions. Amplification of the cDNA product was performed using GAPDH as an internal control with the Revert Aid First Strand cDNA Synthesis Kit (Thermo Scientific, Thermo Electron Co., MA, USA) and the 7500 software (Applied Biosystems, Foster City, CA, USA). Primer sequences were as follows: NRP2: 5′-CATCTCGGCTTTTGCAGGG-3′ (sense), 5′-AGTGCGAGCCACGGTCTTG-3′(antisense); GAPDH: 5′-CAAGGTCATCCATGACAACTTTG-3′ (sense), 5′-GTCCACCACCCTGTTGCTGTAG-3′ (antisense); miR-196a-3p: 5′- CGGCAACAAGAAACUGC CUGAG-3′ (sense), 5′-CAGGCAGUUUCUUGUUGCCG UU-3′ (antisense); U6: 5′-GCTTCGGCAGCACATATA CTAAAAT-3′ (sense), 5′-CGCTTCACGAATTTGCGTGT CAT-3′ (antisense). PCR conditions for mRNA were as follows: activation, 10 min at 95°C; denaturation, 15 s at 95°C; extension, 60 s at 60°C. For miRNAs, reverse transcription and amplification were performed with the PCR system (LongGene, Hangzhou, China) with U6 small nuclear RNA as an internal control. PCR conditions were as follows: activation, 10 min at 95°C; denaturation, 15 s at 95°C; extension, 60 s at 60°C.

### MTT assay

Following transfection, 5000 cells were plated in 96-well plates in 10% FBS and stimulated with 5 ng/ml TGF-β for 24 h. Cells were then incubated for 4 h in 20 μl 3-(4,5-dimethylthiazol-2-yl)-2,5-diphenyltetrazolium bromide (MTT) solution (5 mg/ml MTT powder in phosphate buffer saline(PBS) at 37°C in a humidified atmosphere containing 5% CO2. The MTT solution was removed to incubate the cells in 200 μl DMSO (Sigma, St. Louis, MO, USA). Microplate reader(Thermo Scientific, Thermo Electron Co., MA, USA) was used to determine OD. Absorbance was read at 570 nm.

### Protein extraction and western blot analysis

Cells were transfected with or without siRNA or mimics and treated with or without 5 ng/ml TGF-β for 48h. Total proteins were extracted with radioimmune precipitation buffer containing 1% Triton (1% Triton X-100, 1% sodium deoxycholate, 150 mM sodium chloride, 50 mM Tris, pH 7.2, 0.1% sodium dodecyl sulfate(SDS), 5 mM ethytlene diamine tetraacetic acid (EDTA), pH 8.0, supplemented with 5 μg/ml aprotinin, 1 μg/ml pepstatin, 2 μg/ml leupeptin, 1 mM phenylmethanesulfonyl fluoride (PMSF). Total cell extracts were separated by SDS- polyacrylamide gel electrophoresis(PAGE), semidry transferred to a 0.45-μm polyvinylidene fluoride (PVDF) membrane, and incubated overnight with primary antibodies at 4°C. Immunoreactivity was revealed by ChemiScope5200 chemiluminescence imaging system. The primary antibodies of rabbit anti-NRP2, mouse anti-β-actin were purchased from Abcam, Cambridge, MA, USA.

### Invasion and motility assays

For invasion assays, 1.0 × 10^5^ cells were seeded in a Matrigel-coated chamber with 8.0 mm pores(Corning Inc., NY, USA). For motility assays, 5.0 ×10^4^ cells were plated on uncoated membranes with 8.0 mm pores (Corning Inc.). Cells were seeded in serum-free medium and translocated toward complete growth medium for 24 h.

### Luciferase reporter assays

The MDA-MB-231 cells were seeded onto 24-well plates (5.0×104cells/well) and allowed to adhere overnight. The following day, the cells were transiently transfected using transfection reagent using Lipofectamine 2000 reagent (Invitrogen) with pGL3–X-baI (Promega) and 100 ng/well psi-Check2 luciferase reporter (Promega) that housed the 3′UTR sequence that contained either a wild-type or mutant version of the NRP2 seed sequence and transfected into MDA-MB-231 cells in the presence of a miR-196a-3p mimic or its control. The Renilla luciferase activities were measured by the Luciferase Reporter System (Promega, Madison, WI, USA) at 2 days after transfection.

### Tumor development and growth assays *in vivo*

Twenty athymic nude mice (Model Animal Research Center of Nanjing University, Nanjing, China), 4–6 weeks old, were randomly assigned into groups. MDA-MB-231 cells (5 × 10^6^) were injected into the right mammary fat pad *in situ*. When the xenograft tumors formed by the MDA-MB-231 cells reached 50 mm^3^, miR-196a-3p and control agomir (1 nmol/injection, three times per week) were injected into the tumors. Tumor volume was calculated as 0.5 × x^2^ × y where x is the tumor width and y is the tumor length. After the last *in vivo* optical imaging, all mice were sacrificed for further analysis. All experimental protocols were approved by the Institutional Animal Care and Use Committee of Soochow University.

### Metastasis assays in nude mice

MDA-MB-231 cells (1 ×10^6^) were injected into the tail vein of athymic nude mice. miR-196a-3p and control agomir were injected (5 nmol/injection, three times per week). Mice became moribund and were sacrificed after pulmonary perfusion 4 weeks later. The number of tumor nodules formed on the lung surface was counted. Lungs were excised and fixed in 10% formaldehyde, after which 0.2 mm paraffin-embedded sections were prepared and stained with hematoxylin and eosin.

### Immunostaining

For NRP2 immunostaining of human primary tumors, freshly frozen samples of human primary breast tumors were sectioned, fixed in acetone, and immunostained with anti-NRP2 antibody, anti Ki-67 antibody (Abcam, Cambridge, MA, USA). The samples were incubated in biotinylated anti-rabbit secondary antibody (Beyotime, Shanghai, China) followed by 3,3′-diaminobenzidine tetrahydrochloride (DAB) (Beyotime). The slides were counterstained with hematoxylin.

### Statistical analyses

The software, SPSS Version 16.0 for Windows (SPSS Inc, Chicago, IL, USA), was used for analysis. The Student's t test and chi-square test were used for comparisons.

Survival curves were plotted by the Kaplan–Meier method and compared by the log-rank test. The survival data were evaluated by univariate and multivariate Cox regression. Differences were considered significant when *P* <0.05.

## CONCLUSION

In summary, we showed that miR-196a-3p is stimulated by TGF-β1 and contributes to cell metastasis by targeting NRP2 in breast cancer. miR-196a-3p is expressed at low levels in breast cancer tissues, and prevent the expression of miR-196a-3p was significantly associated with the progression of breast cancer. The TGF-β/ miR-196a-3p/ NRP2 axis could provide further insight into the pathogenesis of breast cancer, and miR-196a-3p might be a potential diagnostic and therapeutic target for the metastasis of breast cancer.

## SUPPLEMENTARY MATERIALS FIGURES


