# Supplementary material for: Transforming growth factor-β1 promotes breast cancer metastasis by downregulating miR-196a-3p expression
Source: Oncotarget. 2017 Mar 17;8(30):49110–22. doi: 10.18632/oncotarget.16308 (PMC5564753; doi:10.18632/oncotarget.16308)
Supplement: Supplementary file 1 [file oncotarget-08-49110-s001.pdf]

Transforming growth factor-β1 promotes breast cancer metastasis by downregulating miR-196a-3p expression

SUPPLEMENTARY MATERIALS

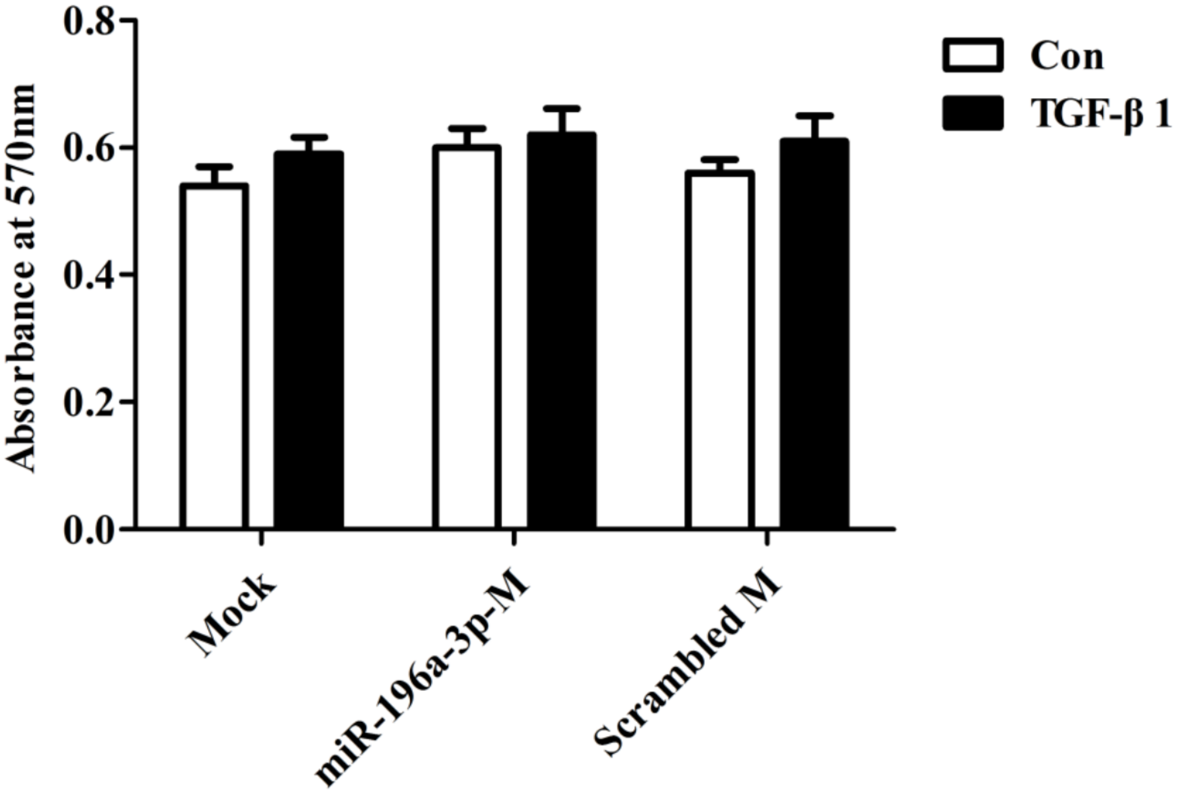

Supplementary Figure 1: Overexpression of miR-196a-3p did not change the proliferative properties of breast cancer cells examined by MTT.

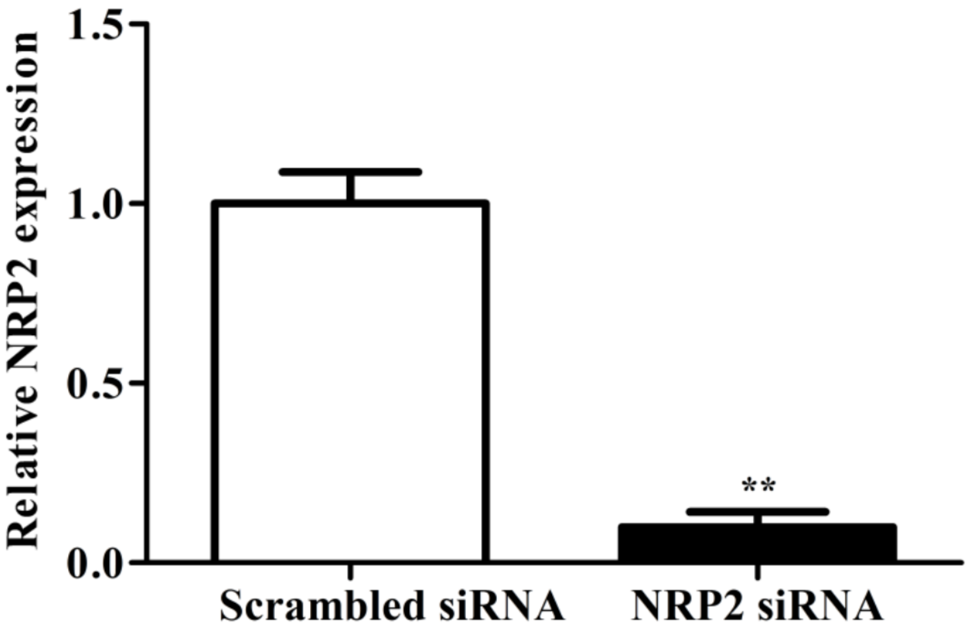

Supplementary Figure 2: Effect of NRP2 siRNA on the expression of NRP2 at the mRNA level. NRP2 siRNA (80 nM) was transfected into MDA-MB-231, NRP2 mRNA levels were measured by qRT-PCR.

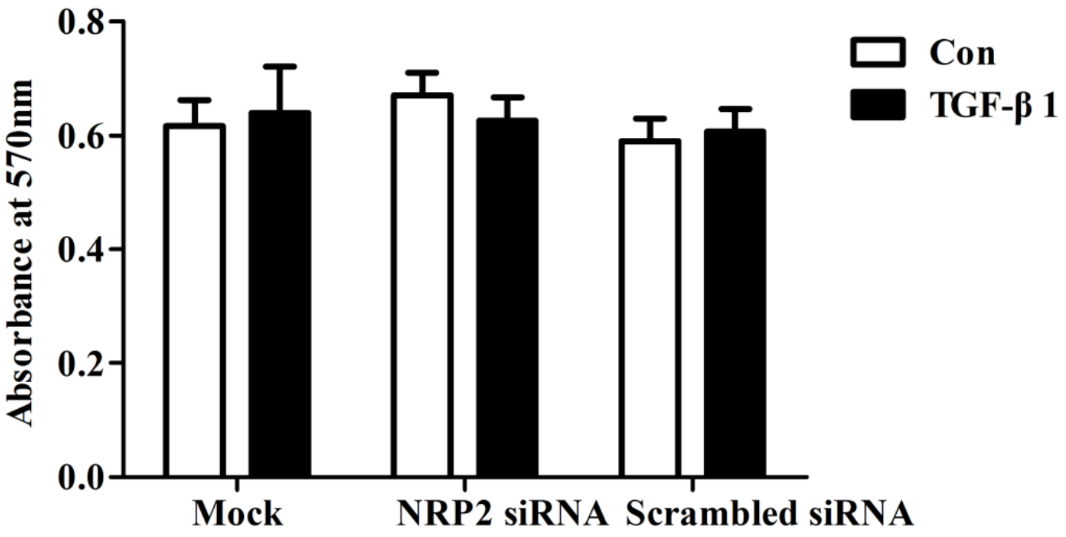

Supplementary Figure 3: Effects of NRP2 siRNA on the proliferation of MDA-MB-231 cells analyzed by the MTT assay. MDA-MB-231 cells were transfected with 80 nM siRNA and siRNA negative controls, and an MTT assay was performed at 72 h after transfection. Cells were stimulated with or without 5ng/ml TGF-β for 24 h.
